# The Degradation Mechanism of Toxic Atractyloside in Herbal Medicines by Decoction

**DOI:** 10.3390/molecules18022018

**Published:** 2013-02-05

**Authors:** Liang-Yu Chen, Anren Hu, Chih-Jui Chang

**Affiliations:** 1 Department of Biotechnology, Ming-Chuan University, Guishan, Taoyuan 333, Taiwan; 2 Department of Laboratory Medicine and Biotechnology, Tzu-Chi University, Hualien 970, Taiwan; 3 Department of Molecular Biology and Human Genetics, Tzu-Chi University, Hualien 970, Taiwan

**Keywords:** atractyloside, decoction, herbal medicines

## Abstract

Atractyloside (ATR) is found in many *Asteraceae* plants that are commonly used as medicinal herbs in China and other eastern Asian countries. ATR binds specifically to the adenine nucleotide translocator in the inner mitochondrial membrane and competitively inhibits ADP and ATP transport. The toxicity of ATR in medical herbs can be reduced by hydrothermal processing, but the mechanisms of ATR degradation are not well understood. In this study, GC-MS coupled with SPE and TMS derivatisation was used to detect ATR levels in traditional Chinese medicinal herbs. Our results suggest that ATR molecules were disrupted by decomposition, hydrolysis and saponification after heating with water (decoction) for a long period of time. Hydrothermal processing could decompose the endogenous toxic compounds and also facilitate the detoxification of raw materials used in the Chinese medicine industry.

## 1. Introduction

Atractyloside (ATR) is one of a group of diterpenoid glycosides. ATR binds specifically to the adenine nucleotide translocator in the inner mitochondrial membrane and competitively inhibits ADP and ATP transport [1]. The toxicity and biochemistry of ATRs have been reviewed [2,3]. ATR is found in many *Asteraceae* plants (*Atractylis*, *Wedelia*, and *Xanthuim* species) that are commonly used as medical herbs in Chinese and other East Asian civilizations [2,4,5,6]. There is a predominant view in Traditional Chinese Medicine (TCM) that herbal medicines are harmless and free of side effects because they come from natural sources. Pharmacological clinical studies on humans showed that dried rhizomes of *A. macrocephala* exerted a diuretic effect in patients with oedema, but not in normal subjects [7,8]. It has also been shown to be liver protective and a mild hypoglycaemic agent [9,10]. *A. lancea* is mainly used for the treatment of indigestion and stomach disorders [11,12]. *Xanthii Fructus* (Cang-Er-Zi) is the dried fruit of *Xanthium strumarium* L. and is used to treat sinusitis, headache and skin pruritus [6]. However, there have been certain cases of hepatic injury and even deaths associated with these medical herbs [13,14,15]. The safe and effective use of medicinal herbs has therefore been identified as a research priority [16,17]. 

**Figure 1:**
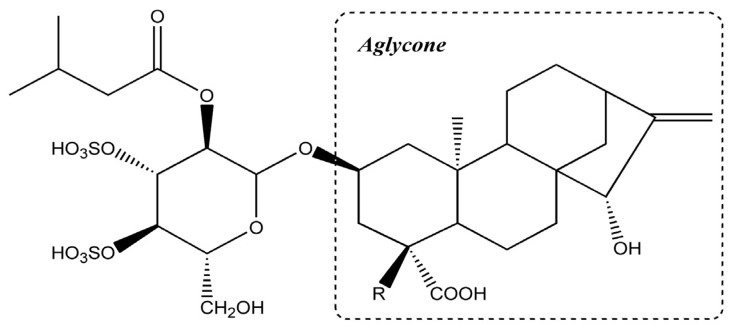
The chemical structures of atractyloside (R = H) and carboxyatractyloside (R = COOH).

The toxicity and chemical profiles of ATRs are highly dependent on the variations in both species and processing [2,18]. Interest in these compounds was stimulated by the high toxicity of the sulphated glucoside and aglycone, which has been responsible for many deadly poisonings [3,19,20]. 4-carboxyatractyloside (CATR), which is more toxic than ATR, is found in fresh, but not dried plants because it is decarboxylated to ATR during ageing or desiccation [2,21]. The structures of ATR and its analogue CATR are illustrated in Figure 1.

Our previous studies suggested that the cytotoxicity of the herbs was attenuated after hydrothermal processing (also called decoction), and we also demonstrated molecular degradation of ATR using mass spectral analysis [22], but the complete mechanism of ATR toxicity is still not fully understood.

In clinical practice, crude herbal medicines are prepared (infused and extracted with hot water) before ingestion of the TCMs. After ingestion, the TCM extracts are likely to be breaking down by action of the gastric juice, a strongly acid and colorless fluid. Therefore, to mimic the process of extraction and digestion of TCM extracts, we analyzed the ATR degradation mechanism under two conditions (temp and pH) in this study. We used a modified GC/MS method for detecting ATR in TCMs and characterised the kinetic degradation of ATR as a possible mechanism for reducing its toxicity. Based on our results, hydrothermal processing could decompose the endogenous toxic compounds and also facilitate the detoxification of raw materials used in the Chinese medicine industry. In addition, acidification could also assist in the degradation, stimulating a detoxification pathway by the action of gastric acid secretions in the stomach. The results from this study will help us to assess the safety and risks of medicinal herbs in the pharmaceutical industry and ethnopharmacology [23,24].

## 2. Results and Discussion

### 2.1. Quantitation of ATR in Herbs

The results of evaluation of ATR levels in herbs are listed in Table 1. *A. gummifera* L. and *Calliepis laureola* DC are used in traditional medicine by people of the Mediterranean regions and South Africa, respectively. The high levels of ATR in two TCM herbs (*Atractylodes lancea and Atractylodes macrocephala*) were determined and compared to other *Asteraceae* plants. Additionally, *Xanthii Fructus* is considered to be acutely toxic because it contains CATR rather than ATR. There are genetic, histological, environmental, and time of harvest factors that influence the ATR content of botanical samples. Our results specified that, due to the presence of toxic materials, TCMs should be collected and stored for an extended period of time before use, because the level of toxic compounds decreases with ageing. Furthermore, the toxic molecule could be degraded by the decocting used in the ethno pharmaceutical procedure.

### 2.2. Cytotoxicity of Herb Extracts and Effects of Decoction 

**Figure 2:**
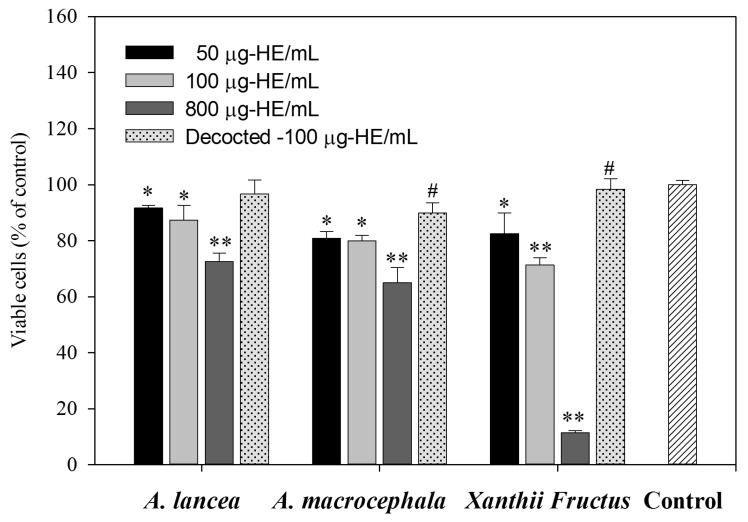
Viability was assessed using an MTT assay after 48 h treatment with the indicated agents. Media only was used as the control. Values represent means and standard deviation of 4 independent experiments. *, *p* < 0.05; **, *p* < 0.01, control versus HEs. # indicates a significant different between the HE treatments and decocted 100 μg/mL herbal extract, *p* < 0.05.

To evaluate the cytotoxicity of the herbal extracts (HEs), cells were treated with various concentrations of HE (50, 100, or 800 μg/mL). The method for preparing the HEs is described in Section 3.2. The resultant cell viability was determined by MTT assay after treatment for 48 h. As shown in Figure 2, cell viability was significantly decreased by 800 μg/mL HE treatment. If we only consider the amount of ATR in HEs, the cytotoxicity of ATR was observed at the relatively low dosage (about 1 μg/mL). Moreover, the *Xanthii Fructus* extract reduced cell viability in a dose-dependent manner. This result implied that higher levels of intensely toxic compounds could be extracted by water from *Xanthii Fructus* than from the dried rhizomes of the *Atractylis* species. 

Decoction is a very convenient process when applied to TCMs. To analyse the effect of hydrothermal processing, aqueous extracts of the three herbs were also heated to boiling for 3 h. The cytotoxicity of the HEs at 100 μg/mL was then evaluated by MTT assay. Interestingly, the toxicity of the herbal extracts was reduced significantly. According to literature reports [3], many endogenous toxins are unstable under conditions of high temperature, oxidation, or light irradiation [25]. Our results also demonstrate the effect of decocting the 100 μg/mL HEs of *A. macrocephala* and *Xanthii Fructus* on retaining cell viability. This suggests that appropriate processing could attenuate the toxicity of crude herbs and this is likely to be through the degradation of ATR.

### 2.3. Hydrolysis of ATR and Mass Spectral Interpretations 

In ethnopharmacology, natural materials are usually formulated and processed before their use as medicines. However, evaluation of toxic compound stability in processing is very difficult because of the complex reactions of the materials [26,27]. Pure ATR and phosphate buffers were used to avoid interference and optimise experimental conditions.

**Figure 3:**
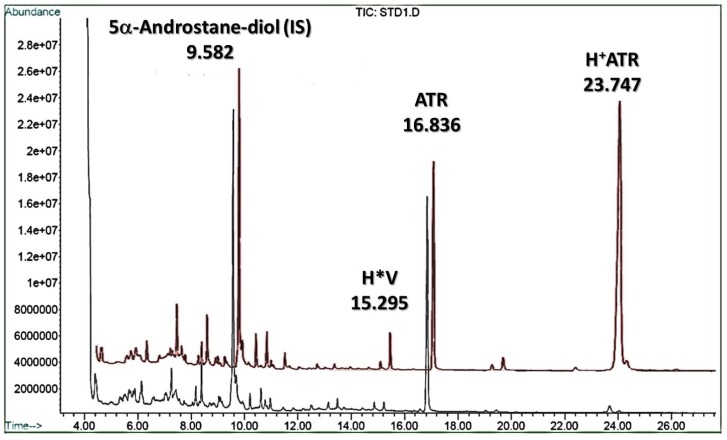
Chromatograms obtained from the TMS derivatives of the hydrolyzed ATR (in red) with the spiked ATR of 100 μg/mL and the internal standard (IS) for comparison.

The GC chromatograms obtained from the TMS derivatives of ATR standards demonstrated that degradation of ATR could generate multiple products during hydrothermal processing. As shown in Figure 3, two new peaks were observed in the chromatogram of acidified and 120 min hydrothermally treated ATR. The major peak (retention time of 23.747 min) was designated as H^+^ATR. The other peak at 15.295 min was little increased and designated as H*V.

**Figure 4:**
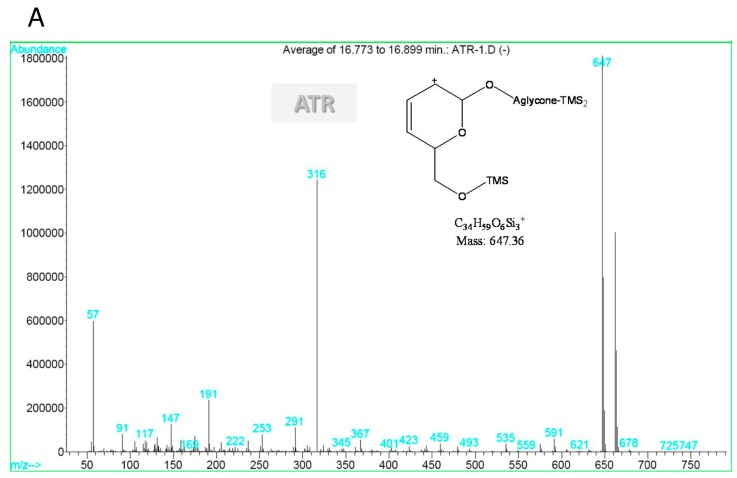

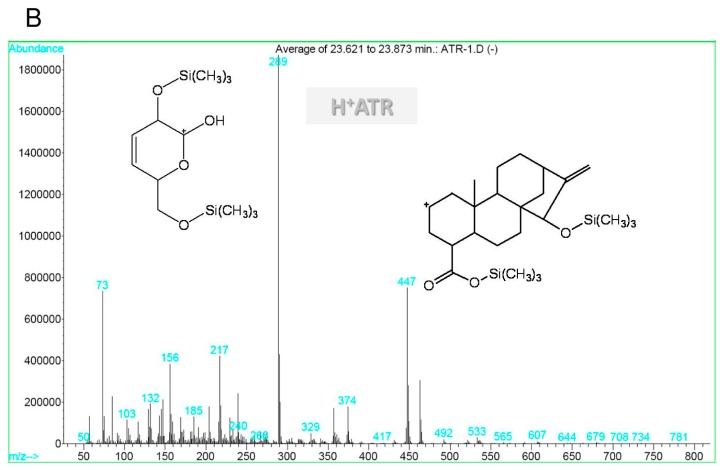
Mass spectra of ATR (**A**) and H+ATR (**B**).

The mass spectrum of ATR (retention time of 16.836 min) was characterised by ion fragments at *m/z* 316, 647, and 663 in Figure 4A. The molecular structure of ATR derivatives should contain at least one sulphate group. The mass spectrum of H^+^ATR was characterised by ion fragments at *m/z* 289 and 447 in Figure 4B.

In a previous study to detect acidified ATR in a patient poisoned with *Callipepis laureola*, the organic solvent ethyl acetate was used to extract lipophilic compounds [19]. However, the acidification of sonicated extract would transform isovalerate ester to acetate ester in ATR. The hydrolysis of isovalerate and sulphate groups was increased by acidification and hydrothermal processing. Here, we used a solid phase extraction (SPE) technique to avoid any transesterification reactions such as biodiesel production.

### 2.4. Degradation of ATR by Hydrothermal Processing

**Figure 5:**
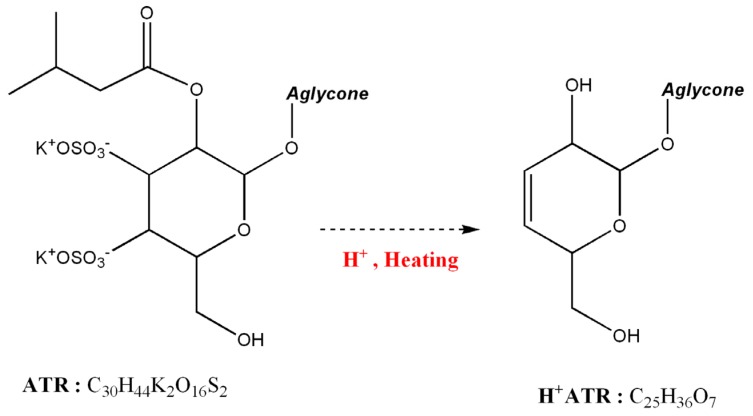
The degradation mechanism of ATR salt by acidification and heating.

Based on the mass spectrum (Figure 4B), the sulphate group could not be observed due to the hydrothermal treatment. Additionally, the structure of the TMS derivative of H^+^ATR should have a new hydroxyl group from the hydrolysis of the isovalerate group in the glycoside moiety. Also, the H*V peak in the chromatograms (in Figure 3) is considered as a TMS derivative of isovaleric acid. Therefore, we suggest that the sulphate groups and the isovalerate ester are highly associated with ATR degradation, as shown in Figure 5. 

**Figure 6:**
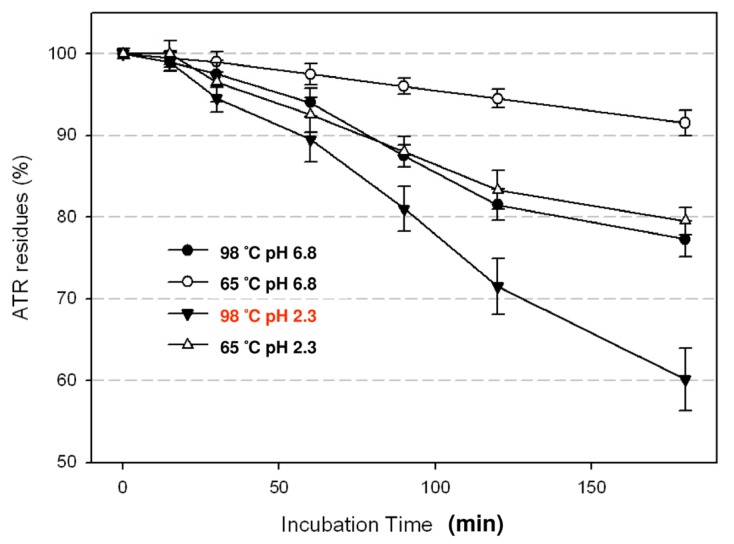
The degradation of ATR by acidification and heating. After acidification and heating for 120 min, the residue of ATR is decreased significantly (the *p* value is less 0.05).

It is difficult to dissect the detailed mechanisms of ATR degradation in herbal extracts, especially in high concentrations. The components are complicated and aggregation is always a problem. Besides, at higher concentrations of herbal extracts, we cannot rule out the possibility that any cytotoxic effects might due to other components in the extracts. In this study, we only focused on the degradation mechanisms of ATR during herbal medicine preparation. Spiked HEs were used to mimic the process of extraction. To reduce perturbation from other components in HEs and to amplify the ATR signal, we added 100 μg/mL of ATR to 0.4% of herbal extracts (diluted from 4% HE stock). The time course of ATR degradation by hydrothermal treatment is shown in Figure 6. At high and moderate temperatures, degradation of ATR was observed and this increased with incubation time. Acidification also enhanced the efficiency of degradation. The use of SPE in the sample preparation procedure could avoid the esterification errors derived from the traditional method with liquid-liquid extractions. The performance of the GC/MS method with SPE was validated by previous studies, which reported a good sensitivity and the 87%–103% recovery of HE spiked 100 μg/mL ATR [22]. 

Approximately 40% of ATR could be removed by hydrothermal processing at 98 °C (without boiling) and pH 2.3 for 2 h. Detoxification resulted from decomposition, hydrolysis and saponification of toxic analytes, which reacted in an aqueous solution of a natural mixture. The effects of hydrothermal processing could explain why reports of intoxication are rare from brewing and drinking herbs in TCMs.

## 3. Experimental 

### 3.1. Chemicals, Reagents and Apparatuses

The potassium salt of atractyloside, 5α-androstane-3β,17β-diol (as internal standard) and the derivative reagent trimethylsilyl imidazole (TMSI) were purchased from Sigma Chemical-Aldrich (St. Louis, MO, USA). Ethyl acetate, methanol and pyridine (Silylation grade) were obtained from Merck (Darmstadt, Germany). All other chemicals were of analytical grade and used without further purification. Water was purified using Millipore Milli-Q water filtration system (Millipore, Bedford, MA, USA). The standard solutions of ATR were 100 and 1,000 μg/mL and were stored in the dark at −20 °C. A 12-port vacuum manifold and HyperSep C18 SPE cartridge (200 mg bed weight per 3 mL volume) were used for extractions and were purchased from Thermo Scientific Corp (Folsom, CA, USA).

### 3.2. Sample Preparation and Aqueous Infusion

*Xanthii Fructus* and the rhizomes of A. macrocephala and A. lancea were collected from the wilderness in Taiwan. These fresh samples were identified by morphological classification and genetic polymorphisms by Kuo-Chieh Ho, Institute of Plant Biology, National Taiwan University, then freeze-dried and weighed. The voucher sample was deposited in Dr. Liang-Yu Chen lab, Department of Biotechnology, Ming-Chuan University. *Atractylode lancea:* No. 114, *Atractylode macrocephala*: No. 115, *Xanthii Fructus*: No. 116.

The dried botanical samples were pulverised to powder before the infusion process (water extraction). Four grams of pulverised powder was sonicated for 30 s, extracted with 40 mL of water by stirring at room temperature for 30 min, and then centrifuged at 1,500 × *g* for 5 min. The extraction was repeated three times. The extracts were collected and filtered through a 0.45 μm PVDF filter, concentrated with a vacuum evaporator and freeze-dried. Each sample was re-dissolved in 100 mL of water and designated as the stock solution of 4% herbal extract which was used subsequently to assess the degradation of ATR.

For MTT assay and experiments quantitating ATR in herbs experiments, 500 g of pulverised powder was extracted in 500 mL of water by stirring at room temperature for 1 h. The extraction was repeated three times. The extracts were collected and filtered through a 0.45 μm PVDF filter, concentrated with a vacuum evaporator and freeze-dried. The powder was dissolved in media to make different concentrations (50, 100 and 800 μL/mL) of herbal extracts for experiments in Section 3.3.

### 3.3. MTT Assay

The human cervical cancer cell line *HeLa* was cultured in DMEM supplemented with 10% foetal bovine serum (Hyclone; Logan, UT, USA) at 37 °C with 5% CO_2_ incubation. Cells were cultured in 96-well plates and treated with 100 μL of different concentrations of herbal extracts (50, 100 and 800 μL/mL) for 48 h. In addition, cells were treated with 100 μL/mL herbal extracts that had been processed hydrothermally by boiling for 3 h. Following incubation, the medium in each well was exchanged, and the cells were incubated with 40 μL of MTT solution (5 mg/mL) for 4 h. Next, 250 μL of DMSO was added to extract the MTT formazan. The reaction products were measured at 540 nm by a 96-well plate ELISA reader (GE Healthcare, Fremont, CA, USA) and the absorbance values used to calculate the % viability cells relative to the control. In order to determine whether there were significant differences in cell viability a T-test was performed.

### 3.4. Stability of ATR during Decoction

The spiked HEs were prepared by adding 100 μg/mL of ATR to 0.4% of herbal extracts (diluted from 4% HE stock). One aliquot was left untreated and stored at ambient temperature (approximately 22 °C) as a control. Five 200 mL aliquots of 100 μg/mL ATR spiked HE of *A. macrocephala* were prepared to evaluate the kinetic stability of ATR by heating and acidification. Samples of spiked HE were adjusted to pH 6.8 or pH 2.3 in 100 mM phosphate buffered saline (PBS), and the solutions were incubated at 98 °C or 65 °C, respectively. Three 2 mL samples were collected at 15, 30, 60, 90, 120, 300 min. The samples were then extracted using SPE and derivatised for GC/MS analysis.

### 3.5. Extraction and GC-MS Analysis

Three 2.0 mL aliquots of the solutions collected in Section 3.4 were transferred into 10 mL polypropylene centrifuge tubes and acidified with hydrochloric acid (2 mL, 2.0 N). After vortexing, samples were centrifuged at 1,500 × *g* for 5 min. Cartridges were conditioned by deionised water after methanol washing for solid phase extraction, and two consecutive elution (2 mL each) were performed with a mixture of methanol (80:20, v/v) containing 2% ammonium hydroxide. The extracts were then evaporated to dryness at 40 °C under a stream of nitrogen.

The dry residues were resuspended in 0.5 mL of methanol and transferred to derivatising vials. After the solvent was again evaporated to dryness with nitrogen, 200 μL of pyridine and TMSI was added. The derivatisation was performed at 100 °C for 2 h before GC-MS analysis.

Analyses were performed with a HP-6890 gas chromatograph and a HP-5973 mass-selective detector (MSD) from Agilent Technologies (Palo Alto, CA, USA) equipped with a capillary gas chromatography column DB-1MS (30 m × 0.25 mm I.D., 0.25 μm film thickness) (J&W Scientific, Folsom, CA, USA). The injection volume was 2 μL at splitless mode, and the inlet temperature was 250 °C. The carrier gas was helium at a flow rate of 1 mL/min, and the oven temperature was programmed as follows: initial temperature at 210 °C for 3 min and increased at 10 °C/min up to 270 °C, then increased at 30 °C/min until 310 °C, and then held for 15 min. The analysis was performed in the EI mode, and the ionisation voltage was fixed at 70 eV. The scan acquisition (*m*/*z* 50–800) of MSD was performed by the HP chemistation software. Quantitative data were calculated by the internal standard method based on integrated area of peaks in selected ion monitoring (SIM) mode.

## 4. Conclusions

It is proposed that ATR makes a major contribution to the cytotoxic properties of the species *Atractylis.* GC-MS with SPE and TMS derivatisation allows for the detection of ATR in traditional Chinese medicinal herbs. This method is ideal for general screening through forensic analysis or as a quantitative evaluation of hazard analysis and critical control point (HACCP) in pharmaceutical safety. The structure of ATR molecules was disrupted by heating with water (decoction) for a long period of time. This study validates the degradation of ATR as a possible mechanism for reducing its toxicity. Hydrothermal processing could also facilitate the detoxification of the raw materials used in the Chinese medicine industry.

## Figures and Tables

**Table 1:** ATR content of plants.

Specimen	ATR ± s.d. (μg/g) ^a^	Reference
*Atractylode lancea* (n = 3	8980 ± 148	This study
*Atractylode macrocephala* (n = 3)	9230 ± 175	This study
*Xanthii Fructus* (n = 3)	2570 ± 153	This study
*Calliepis laureola*	N.D.	[21]
*Atractylode gummifera*	0.12%–1.57%	[3]

^a^ Contents of ATR are based on dried weight. ^b^ N.D. represents not detectable, less than the limit of detection (LOD = 3 × s.d.).
